# Ciprofloxacin is an inhibitor of the Mcm2-7 replicative helicase

**DOI:** 10.1042/BSR20130083

**Published:** 2013-10-07

**Authors:** Nicholas Simon, Matthew L. Bochman, Sandlin Seguin, Jeffrey L. Brodsky, William L. Seibel, Anthony Schwacha

**Affiliations:** *Department of Biological Sciences, University of Pittsburgh, Pittsburgh, PA 15260, U.S.A.; †Drug Discovery Center, University of Cincinnati, Cincinnati, OH 45237, U.S.A.; ‡Molecular and Cellular Biochemistry Department, Indiana University, Bloomington, IN 47405, U.S.A.

**Keywords:** AAA+ ATPase, DNA replication, fluoroquinolone, helicase, minichromosome maintenance complex, CI, confidence interval, DCCD, N,N′-dicyclohexylcarbodi-imide, EEDQ, N-ethoxy-carbonyl-2-ethoxy-1,2-dyhydroquinolone, Mcm, minichromosome maintenance protein, Nbf, 4-chloro-7-nitrobenzofurazan, NEM, N-ethylmaleimide, PG, phenylglyoxal, PP, pyridoxal phosphate, SsoMcm, *Sulfolobus solfataricus* Mcm protein, SV40, simian virus 40, TAg, T-antigen, Topo I, topoisomerase I, Topo II, topoisomerase II

## Abstract

Most currently available small molecule inhibitors of DNA replication lack enzymatic specificity, resulting in deleterious side effects during use in cancer chemotherapy and limited experimental usefulness as mechanistic tools to study DNA replication. Towards development of targeted replication inhibitors, we have focused on Mcm2-7 (minichromosome maintenance protein 2–7), a highly conserved helicase and key regulatory component of eukaryotic DNA replication. Unexpectedly we found that the fluoroquinolone antibiotic ciprofloxacin preferentially inhibits Mcm2-7. Ciprofloxacin blocks the DNA helicase activity of Mcm2-7 at concentrations that have little effect on other tested helicases and prevents the proliferation of both yeast and human cells at concentrations similar to those that inhibit DNA unwinding. Moreover, a previously characterized mcm mutant (mcm4chaos3) exhibits increased ciprofloxacin resistance. To identify more potent Mcm2-7 inhibitors, we screened molecules that are structurally related to ciprofloxacin and identified several that compromise the Mcm2-7 helicase activity at lower concentrations. Our results indicate that ciprofloxacin targets Mcm2-7 *in vitro*, and support the feasibility of developing specific quinolone-based inhibitors of Mcm2-7 for therapeutic and experimental applications.

## INTRODUCTION

As cancer cells demonstrate uncontrolled proliferation relative to most non-cancer cells, DNA replication has traditionally been an important target for cancer chemotherapy. Such therapeutics are frequently nonspecific and mutagenic, as they either chemically modify the DNA to block replication fork progression or trap deleterious Topo II (topoisomerase II)/DNA double-strand break intermediates [1]. Not surprisingly, these therapies have multiple toxic side effects (reviewed in [2]). Newer topoisomerase inhibitors, which inhibit the catalytic activity of the enzyme rather than trapping the toxic protein–DNA intermediate, show therapeutic promise [3], suggesting that compounds that specifically inhibit DNA replication enzymatic activity may be better suited as therapeutic agents. Moreover, enzyme inhibitors have had a long and important history in biochemical research, and their use has been an essential avenue to obtain critical mechanistic insight (e.g., the F1 ATPase [4]). As eukaryotic DNA replication is a complex process that is poorly understood at a mechanistic level, the development of targeted small molecule inhibitors of specific replication factors would be of significant research utility.

One potential therapeutic target is the Mcm2-7 (minichromosome maintenance protein 2–7) eukaryotic replicative helicase, a molecular motor that unwinds duplex DNA to generate ssDNA templates for replication. Unlike other replicative helicases, the toroidal Mcm2-7 complex is formed from six distinct and essential subunits, numbered Mcm2 through Mcm7 [5]. Each subunit is an AAA^+^ ATPase, and the unique heterohexameric composition of this helicase is conserved throughout eukaryotic evolution (reviewed in [5]). Consistent with its vital function during DNA replication, Mcm2-7 is a key target of regulation, as its loading is a carefully controlled and limiting feature of replication initiation, whereas its cell cycle-dependent activation is a limiting feature of elongation [6]. The importance of its regulation is demonstrated by the observations that both specific mutations in Mcm2-7 [7] and overexpression of its subunits [8] cause cancer or contribute to tumorigenesis. Despite the potential of helicases as disease targets, a few specific small molecule inhibitors of these enzymes have been identified [9–12]. To date, one compound, heliquinomycin, has been identified that inhibits a non-physiological Mcm subcomplex (Mcm467) [13] and decreases the proliferation of cancer cells *in vitro* [14], further suggesting that Mcm inhibitors may have therapeutic value.

Following examination of amino acid modifiers and small molecule ATPase inhibitors [4,10,11], we found that the commercially available fluoroquinolone antibiotic ciprofloxacin preferentially inhibits the *in vitro* helicase activity of the *Saccharomyces cerevisiae* Mcm2-7 complex. Ciprofloxacin also appears to target Mcm2-7 in cell culture, as it blocks proliferation of both yeast and human cells at concentrations that inhibit the purified enzyme, and a previously studied cancer-causing mutation in Mcm4 confers ciprofloxacin resistance [15]. Additional inhibitors of greater potency were identified among compounds structurally related to ciprofloxacin. Several of these agents exhibited increased selectivity towards Mcm2-7, whereas others had varying specificities against a range of unrelated helicases. These data suggest that (fluoro)quinolone-based compounds may provide a general scaffold for future development of helicase inhibitors with targeted specificity.

## MATERIALS AND METHODS

### Chemicals

Stock solutions of putative inhibitors were made in anhydrous DMSO at either 13 mM (MAL2-11B [11]) or 100 mM [EEDQ (N-ethoxy-carbonyl-2-ethoxy-1,2-dyhydroquinolone; Aldrich), DCCD (N,N′-dicyclohexylcarbodi-imide; Sigma), PP (pyridoxal 5′-phosphate; Fluka), PG (phenylglyoxal; Aldrich), Nbf (4-chloro-7-nitrobenzofurazan; Fluka), ofloxacin (Sigma) and ciprofloxacin (Fluka,>98% pure by HPLC)]. NEM (N-ethylmaleimide, USB) was made as a 1 M stock in absolute ethanol. These stock solutions were stored at −20°C and were stable for at least several months. All compounds were completely soluble at the final assay concentrations except as noted.

For initial small molecule inhibitor screening, a collection of 144 compounds was obtained from the DDC (Drug Discovery Center, University of Cincinnati, Cincinnati, OH) (Supplementary Table S1 available at http://www.bioscirep.org/bsr/033/bsr033e072add.htm). For follow-up experiments on selected inhibitors (Table 1), neat samples of each inhibitor were obtained from DDC or ChemBridge (compounds 924384 and 271327 correspond to ChemBridge 7473736 and 5281925, respectively) and stored as 100 mM stock solutions in DMSO. The purity of these compounds was either established by the manufacturer or was determined by the DDC using mass spectrometry and HPLC analysis and found to be >90–100% in all cases (Table 1).

**Table 1:** Structures and IC_50_ values of select inhibitors DDC-UC, Drug Discovery Center, the University of Cincinnati. ND, not done.

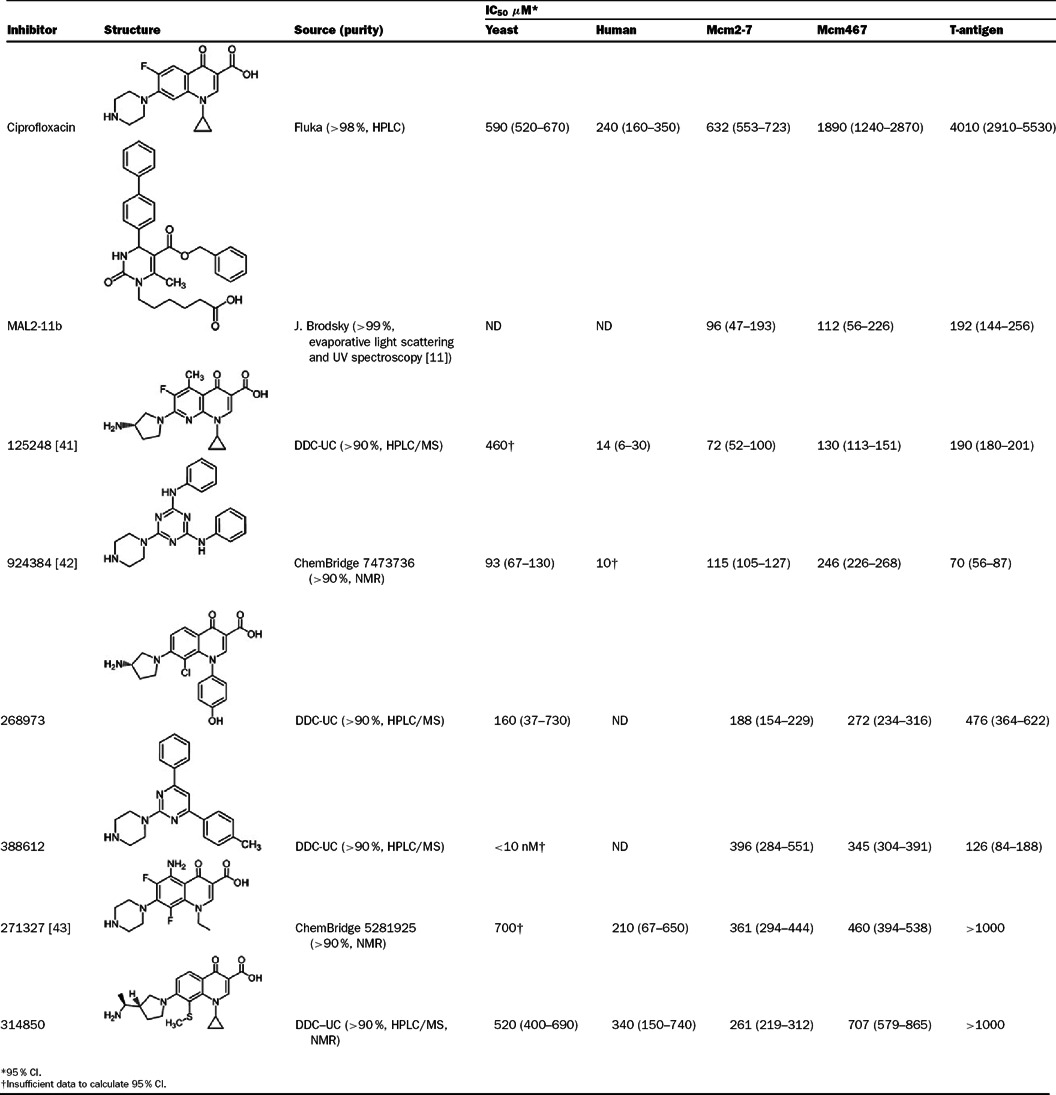

### Proteins

Hexameric *S. cerevisiae* Mcm2-7 and Mcm467 complexes were expressed in baculovirus-infected insect cells, and purified using affinity, gel filtration and ion exchange chromatography as described [16–19]. Gel filtration and western analysis indicates that >50% of the complexes contain either all three (i.e., Mcm467) or all six (i.e., Mcm2-7) of the indicated Mcm subunits. The SV40 (simian virus 40) large TAg (tumour antigen) was purified as previously described [20]. Additional helicases were generously provided by colleagues: the SsoMcm (*Sulfolobus solfataricus* Mcm protein) complex (M. Trakselis, University of Pittsburgh, Pittsburgh, PA); Srs2 (E. Antony, Washington University School of Medicine, St. Louis, MO); T7 gp4 (S. Patel, University of Medicine and Dentistry of New Jersey, Piscataway, NJ); DnaB (K. Marians, Sloan-Kettering, New York, NY) and T4 gp41 (S. Benkovic, Pennsylvania State University, University Park, PA).

### Biochemical assays

Helicase assays were performed essentially as described [16,19]. Synthetic replication forks were prepared by annealing oligos 233 and 235 [IDT (Coralville), oligo 233 5′(T)_40_GGTTGGCCGATCAAGTGCCCAGTCACGACGTTGTAAAACGAGCCC; oligo 235 5′ CACTCGGGCTCGTTTTACAACGTCGTGACTGGGCACTTGATCGGCCAACC(T)_40_] and then filling in the recessed 3′-end with [α32P]dATP and unlabelled dNTPs using Klenow Fragment. Briefly, reactions (6 ml) were performed in 1× helicase assay buffer [20 mM Tris/HCl (pH 7.5), 10 mM MgOAc, 20% (v/v) glycerol, 100 μM EDTA and 5 mM DTT (dithiothreitol)] and contained a final concentration of 1 nM fork substrate, 5 mM ATP, 40 mM creatine phosphate, 16.5 mg/ml creatine kinase, 33 μg/ml BSA. Reactions containing Mcm2-7 and Mcm467 were supplemented with 200 mM potassium glutamate. Reactions containing SsoMcm were incubated at 65°C, those containing T4 gp41 were incubated for 30 min at 37°C, and all other reactions were incubated at 37°C for 1 h. The products were separated by 10% (w/v) native PAGE, the resulting gels dried and the radioactivity quantified using a Fuji FLA-5100 phosphoimager. Irrespective of the protein used, all helicase assays contained equal molar helicase concentrations (100 nM, assuming in all cases that the active helicase form is hexameric). Steady-state ATP hydrolysis was assayed as published [17]. In short, reactions were set up essentially as in the helicase assay, with minor exceptions. A non-radiolabelled DNA fork was used, helicase concentration was 100 nM (hexamer) the total ATP concentration was 500 μM and included ~0.5 μCi of [α32P]ATP, and the ATP regeneration system was omitted. Reactions were incubated for 1 h at 37°C and stopped by the addition of SDS. ATP was separated from ADP by PEI (polyethyleneimine) thin layer chromatography, and the ratio of ATP:ADP was quantified using a Fuji FLA-5100 phosphoimager. Based on our prior work [17], conditions were established to ensure that the results shown are within the linear range of the assay. Protein–ssDNA binding was determined with a double filter-binding assay using an ssDNA probe (oligo 826, 5′TGTCTAATCCCGAAAGGCCCTGCCACTGAAATCAACACCTAAAGCATTGA) that was 5′-radiolabelled using T4 polynucleotide kinase and [γ32P]ATP [16]. For the double filter-binding assay, the helicase concentration was 150 nM (hexamer) and the ssDNA concentration was 4 nM. For all biochemical assays, helicases were preincubated with inhibitors for 20 min at 37°C unless otherwise indicated.

Topo I (Topoisomerase I) assays were performed as described [21]. Reactions (10 μl) contained 50 mM Tris/HCl (pH 8), 1 mM EDTA, 1 mM DTT, 20% (v/v) glycerol and 50 mM NaCl. pUC19 (50 ng; NEB) was incubated at 37°C for 2.5 h with 4 units of Wheat Germ Topo I (Promega). Inhibitors were added at the indicated concentrations at either *t*=0 or 90 min as described in the figure legends. Following incubation, topoisomers were separated via gel electrophoresis on a 1.0% (w/v) agarose gel for 2 h at 8 V/cm in TAE (Tris/acetate/EDTA) buffer. After electrophoresis, the gel was stained with ethidium bromide and imaged with a Fuji LAS-3000. In all of the above assays, dilutions of the test compound were made with Milli-Q H_2_O and DMSO such that the final concentration of DMSO in the biochemical assays was 1% (v/v), and the reported activity was normalized to solvent controls.

### Growth assay

*S. cerevisiae* growth inhibition was quantified using an established 96-well assay [22]. Two isogenic W303 tester strains were used (construction details available upon request): UPY675 (*matA*, *ade2-1*, *ura3-1*, *his3-11*,*15*, *trp1-1*, *leu2-3*,*112*, *can1-100*, *bar1*::*hisG*, *Δerg6*::*kanMX*) and UPY1056 (isogenic to UPY675 but containing *mcm4chaos3*). Overnight yeast cultures were grown in YPD medium (1% (w/v) yeast extract/2% (w/v) peptone/2% (w/v) glucose), diluted to 0.05 *A*_600_, grown to an *A*_600_ of 0.1–0.15, divided into aliquots into a 96-well plate, and then treated with inhibitor titrations. Inhibitors were first diluted in pure DMSO, and then added to wells to a final volume of 100 μl containing 2% (v/v) DMSO. Plates were grown without shaking at 30°C for 24 h. Optical density was quantified at 0 and 24 h with a Bio-tek EL800 Universal Microplate reader. Percent relative growth was determined by calculating the change in optical density over 24 h at each concentration relative to a 2% DMSO control.

### Data analysis

Inhibition and the corresponding 95% CIs (confidence intervals) from both the helicase assays and growth inhibition assays were plotted using GraphPad Prism Version 5.0f for Macintosh. The inhibitor concentrations were converted to Log_10_, and then nonlinear regression was used to fit the data points with a sigmoidal dose–response curve [eqn (1)]

(1) $$y\;=\;y_{\min}\;+\left(\frac{y_{\max}-y_{\min}}{{}_{1+10}\left(\log{\mathrm{IC}}_{50}-x\right)-\mathrm{Hillslope}}\right)$$

where *y*_min_ is the minimum helicase activity, *y*_max_ is the maximum helicase activity, IC_50_ is the effective concentration of inhibitor that decreased helicase activity by 50%, and the Hill Slope describes the steepness of the curve. In all cases, eqn (1) was constrained by subtracting the baseline from the data and normalizing all values to helicase activity in the absence of inhibitor. Thus, *y*_min_ and *y*_max_ were 0 and 100%, respectively. The software was also used to calculate the 95% CIs, the quality of the fit (i.e., *R*^2^), and to determine the extra sum-of-squares *F* test to calculate *P* values to compare the LogIC_50_ values between curves. Differences in values were considered statistically significant when *P*<0.05.

## RESULTS

### Experimental rationale

The goal of this study was to identify compounds that preferentially inhibit Mcm2-7. Prior work has demonstrated that the six *S. cerevisiae* Mcm2-7 ATPase active sites contribute unequally to ATP hydrolysis: three are particularly important for DNA unwinding and contribute the most to ATP turnover, whereas the other three contribute little to bulk ATP hydrolysis and appear to play a regulatory role [17–19]. To identify inhibitors that preferentially target one of these two sets of active sites, each inhibitor was tested on both the Mcm467 complex (an *S. cerevisiae* Mcm subcomplex that demonstrates helicase activity but lacks all of the regulatory sites) and Mcm2-7 (containing both types of active sites [19]). As an additional selectivity test, the DNA unwinding activity of another hexameric AAA^+^ helicase (large TAg) was also examined. Based on these considerations, we defined Mcm regulatory inhibitors as compounds that inhibited DNA unwinding of Mcm2-7 but had a negligible effect on Mcm467 or TAg at similar concentrations. Likewise, we defined Mcm catalytic inhibitors as those that inhibited both Mcm467 and Mcm2-7 but that had little or no effect on TAg.

### Chemical modifiers and small molecule inhibitors that preferentially inhibit Mcm or TAg helicase activity

Initially, we tested the effects of both chemical modifiers and previously studied small molecule inhibitors on the helicase activities of Mcm2-7, Mcm467 and TAg by using an established, gel-based, endpoint DNA unwinding assay [19]. The incubation time of our standard assay (30 min) was doubled to eliminate or reduce the identification of weak inhibitors in the screen but remained in the linear range of the assay.

**Figure 1:**
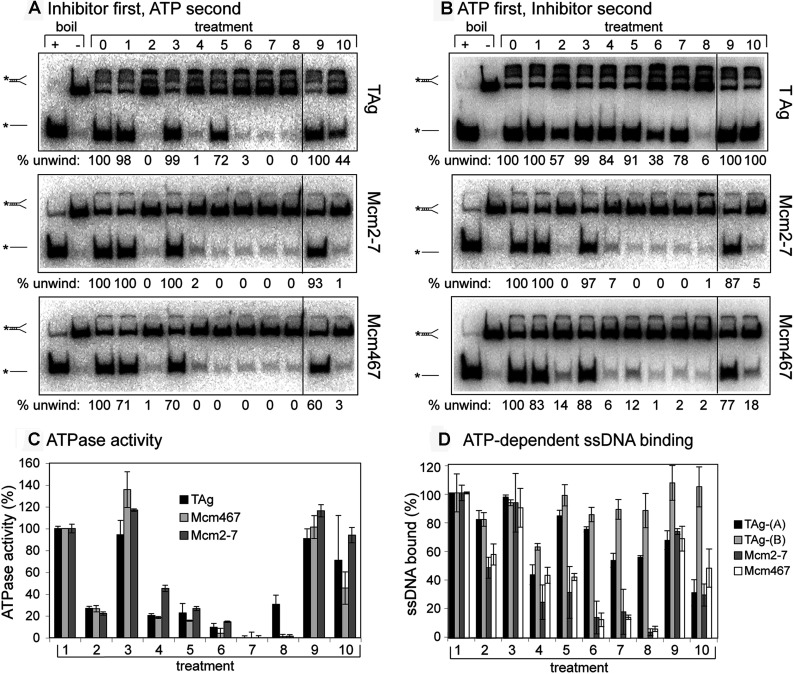
Identification of Mcm small molecule inhibitors (**A**) Inhibition of helicase activity. Helicase assays were conducted as described in the Experimental Procedures, and the indicated proteins (TAg, Mcm2-7, Mcm467) at 100 nM final concentration (of hexameric complexes) were preincubated with the indicated small molecules (treatments 2–10) for 20 min at 37°C prior to addition of ATP and the DNA substrate. For each panel: +, boiled DNA fork; -, intact fork; 0, reconstituted helicase assay without small molecule; 1, standard assay containing 1% (v/v) DMSO. Treatments 2–10 are reconstituted helicase assays additionally containing 1 mM of the following compounds: 2, EEDQ; 3, DCCD; 4, PP; 5, PG; 6, Nbf; 7, NEM; 8, MAL2-11b; 9, ofloxacin; and 10, ciprofloxacin. (**B**) With SV40 TAg, prior ATP preincubation protects against inhibition. This experiment was identical to (**A**), except that the indicated helicase was preincubated with 5 mM ATP for 20 min at 37°C prior to addition of inhibitor and DNA substrate. The discontinuities in these gel images, denoted by a vertical line between treatments 8 and 9, indicates the location where an irrelevant treatment in the assay was electronically removed. (**C**) The small molecules have variable effects on ssDNA binding. Filter binding assays were conducted as described in the Experimental Procedures section using 150 nM of the indicated helicase. For TAg-(A), Mcm2-7 and Mcm467, the indicated helicase was incubated with the small molecule prior to ATP addition as in (**A**); for TAg-(B), TAg was preincubated with ATP prior to small molecule addition as in (**B**). (**D**) Small molecule inhibition of helicase ATPase activity. ATPase activity was assayed as described in the Experimental Procedures section using 100 nM final helicase hexamer concentration. The treatment numbering in (**C**) and (**D**) are identical to those in (**A**). The data in Figure 1C represent the average of ≥2 experiments, and the error bars represent the range or standard deviation, as appropriate. The data in Figure 1(**D**) represent the average of ≥3 experiments, and the error bars represent the S.D.

A variety of amino acid modifiers were initially tested. These chemical probes covalently modify carboxyl groups (carbodiimide derivatives EEDQ and DCCD), guanidyl groups (PG), amino groups (PP), phenol groups (Nbf) and thiol groups (NEM) and have been previously used to study the ATPase active sites in the F1-ATPase (reviewed in [4]). Although most of these amino acid modifiers inhibited all three helicases, DCCD had no effect (Figure 1A, treatment 3), and PG (Figure 1A, treatment 5) preferentially inhibited Mcm2-7 and Mcm467, suggesting the unique role of one or more accessible arginines in the Mcm complexes, possibly the external β-hairpin, a motif that is lacking in SV40 TAg and contains a conserved arginine (see Figure 2 in [5]).

**Figure 2:**
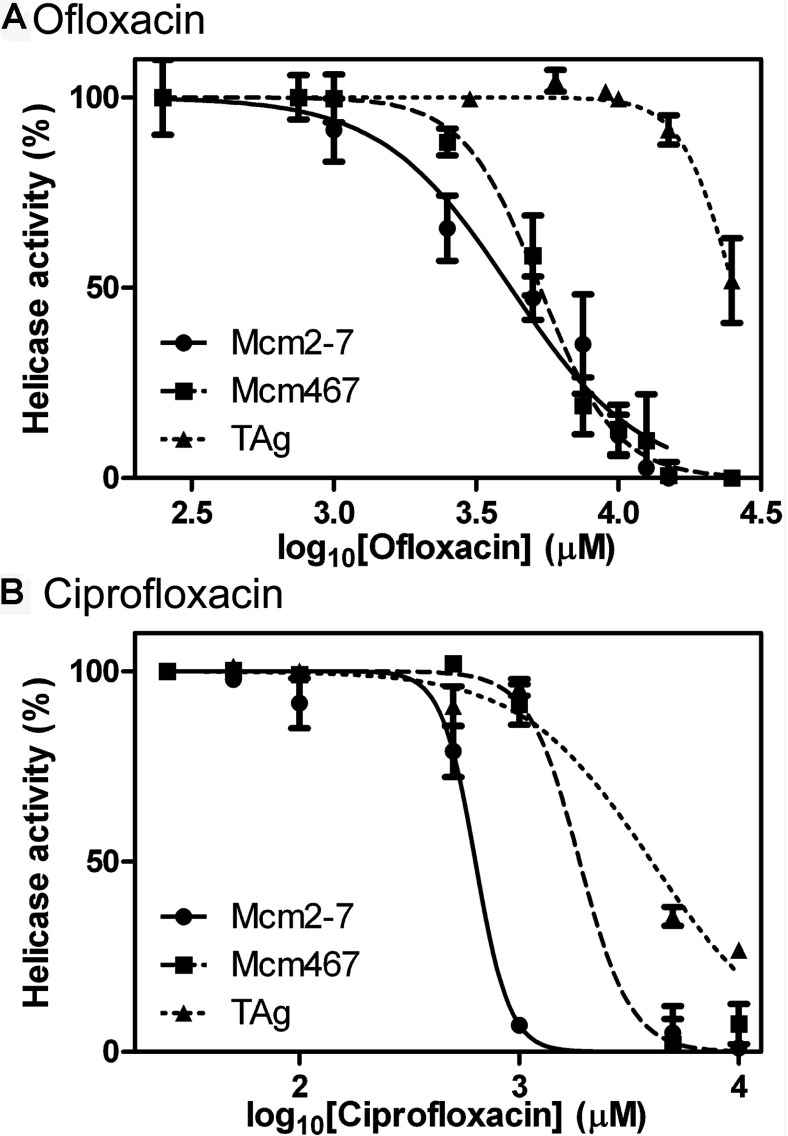
Two fluoroquinolones exhibit preferential inhibition of the Mcm helicases The inhibitor effects of either ofloxacin (**A**) or ciprofloxacin (**B**) were tested on the DNA unwinding activity; the indicated helicases were all used at a final concentration of 100 nM (of complexes) following preincubation with inhibitor. The results were quantified and converted to % activity relative to the respective activity in the absence of inhibitor. All reactions contained 1% solvent (DMSO).

The effects of several previously identified helicase inhibitors were also examined. The pyrimidinone–peptoid hybrid molecule MAL2-11b and the fluoroquinolones ofloxacin and ciprofloxacin have been previously reported to inhibit various TAg-mediated activities [10,11]. MAL2-11b inhibited all three helicases to a similar extent at 1 mM (Figure 1A, treatment 8), but little or no inhibition of TAg helicase activity was observed with 1 mM ciprofloxacin or ofloxacin (Figure 1A, treatments 9 and 10; however, inhibition was observed at higher concentrations, see below). In contrast, 1 mM ciprofloxacin inhibited the helicase activity of both Mcm2-7 and Mcm467 (Figure 1A, treatment 10).

Because TAg subunits oligomerize only in the presence of ATP [23], and ATP preincubation probably causes a conformational change in Mcm2-7 [16,19], we also tested the effects of the potential inhibitors after the proteins were preincubated with ATP (Figure 1B). Although this treatment had essentially no effect on either Mcm complex, it completely or partially protected TAg from all modifiers except Nbf (Figure 1B, treatment 6) and MAL2-11b (Figure 1B, treatment 8), suggesting that at least one effect of the other inhibitors may be to block TAg oligomerization.

Because helicase activity depends on ATP hydrolysis and ssDNA binding, the effects of the chemical modifiers and small molecules on both activities were examined. Using previously established steady-state ATP hydrolysis [17] and ssDNA filter-binding [16] assays, the effect of the same panel of small molecules on each of the three helicases was examined. With the exception of DCCD and ofloxacin, which failed to inhibit helicase activity, most of the remaining treatments severely inhibited the ATPase activities of all three helicases (Figure 1C). These data suggest that the inhibition of DNA unwinding is mediated by compromised function of one or several ATPase active sites. However, these small molecules caused a less severe and variable decrease in TAg ssDNA binding regardless of the order of ATP addition. Conversely, Nbf, NEM and MAL2-11b did inhibit Mcm2-7 and Mcm467 ssDNA binding (Figure 1D, treatments 6–8). Ciprofloxacin stands in sharp contrast: even though it completely inhibited Mcm helicase activity, it had only modest effects on ATP hydrolysis and ssDNA binding of the three helicases (Figures 1C and 1D, treatment 10). Together, these results suggest that ciprofloxacin inhibits a step or steps specifically required for DNA unwinding, possibly through selective inhibition of the Mcm regulatory subunits. This possibility is explored further below.

### Ciprofloxacin demonstrates selectivity towards Mcm2-7

We quantified the IC_50_ values of ofloxacin and ciprofloxacin on Mcm2-7, Mcm467 and TAg helicase activity. We found that very high concentrations of ofloxacin inhibited both Mcm2-7 and Mcm467 with similar IC_50_s (Figure 2A): 4.17 mM (95% CI=3.31–5.26 mM) and 5.29 mM (95% CI=4.92–5.69 mM), respectively, whereas the apparent IC_50_ of ofloxacin for TAg was much higher (>20 mM; Figure 2A). In contrast naladixic acid, the parent quinolone compound for both ciprofloxacin and ofloxacin, had essentially no effect on the activities of the three helicases at any concentration tested (results not shown).

Interestingly, ciprofloxacin inhibited Mcm2-7 at an approximately 3-fold lower concentration than Mcm467 (Mcm2-7 IC_50_=632 μM, 95% CI=552–723 μM; Mcm467 IC_50_=1.89 mM, 95% CI=1.24–2.87 mM, respectively; Figure 2B) and at an approximately 6-fold lower concentration than TAg (IC_50_=4 mM, 95% CI=2.91–5.53 mM). This selectivity of ciprofloxacin for Mcm2-7 relative to TAg supports the proposal that Mcm-specific inhibitors may be found. In addition, the selectivity of ciprofloxacin for Mcm2-7 relative to Mcm467 supports the proposal that active site-specific inhibitors of the Mcm complex can be identified.

### A small molecule library screen for helicase inhibitors

We reasoned that other (fluoro)quinolone derivatives might show enhanced Mcm2-7 specificity at potentially lower inhibitor concentrations. As the fluoroquinolones are used as antibiotics (reviewed in [24]), prior drug discovery efforts have resulted in the synthesis of chemically diverse libraries modeled on key elements found in the basic fluoroquinolone scaffold. Therefore we investigated a 144-compound chemical library that contained either (fluoro)quinolone derivatives or molecules with various substructures found in ciprofloxacin and other marketed quinolones.

This library of 144 compounds was initially screened for inhibition of Mcm2-7, Mcm467 and TAg helicase activity at a final concentration of 1 mM (see Supplementary Table S1 available at http://www.bioscirep.org/bsr/033/bsr033e072add.htm) for chemical structures and a complete list of results. Of the compounds tested, 27 reproducibly inhibited at least one of the three helicases to ≥90%. Both (fluoro)quinolone and triaminotriazine-like inhibitors were identified. Although a wide range of results were obtained, two general conclusions emerged from the data (Supplementary Table S1): (1) Few molecules exhibited robust inhibition of TAg, and those that did (e.g., 924384, 125248 and 486369) also inhibited Mcm2-7 and Mcm467; and (2) many molecules demonstrated at least partial inhibition of Mcm2-7 with little or no inhibition of TAg. Interestingly, although some of the inhibitors appeared to inhibit both Mcm2-7 and Mcm467, the relative strength of this inhibition varied. One agent appeared to act like ciprofloxacin and preferentially inhibited Mcm2-7 (314850), whereas others appear to preferentially inhibit Mcm467 (e.g., 502432 and 502423).

### Select library compounds display greater potency and selectivity than ciprofloxacin

In addition to ciprofloxacin, seven representative compounds from among those described above were chosen for additional study based either upon potency, selectivity, reproducibility, dose-dependent effect and/or availability. Supplementary Figure S1 (available at http://www.bioscirep.org/bsr/033/bsr033e072add.htm) summarizes their effects on the DNA unwinding activity of TAg, Mcm2-7 and Mcm467, again at a final concentration of 1 mM. To provide a quantitative measure of inhibitor affinity and selectivity, fresh samples of known purity (>90%) were obtained for each of the seven inhibitors, and the IC_50_ values for DNA unwinding were determined for all three helicases. In most cases, these compounds were either more potent or more selective than ciprofloxacin (Supplementary Figure S2 and Table S1 available at http://www.bioscirep.org/bsr/033/bsr033e072add.htm). Based on their differential inhibition of the three helicases, the inhibitors were classified into one of two groups:

#### General inhibitors

Inhibitors that had approximately equal effects on all three helicases include MAL2-11b (Figure 1A) and compounds 125248, 924384, 268973 and 388612 (Table 1). Interestingly, unlike any of the (fluoro)quinolones characterized, the triazole 924384 and the structurally related compound 388612 were more effective at inhibiting TAg than either Mcm complex (Table 1). The IC_50_ values for each of these compounds are similar to one another and ranged from ~50 to 400 μM.

#### Mcm-selective inhibitors

Two inhibitors (271327 and 314850) fall into this category. The fluoroquinolone 271327 inhibited both Mcm complexes with an IC50 of ~300–450 μM but had a negligible effect on TAg within the concentration range tested (Table 1). Although the limited solubility of 271327 prevented us from testing higher concentrations, we can conclude that the IC_50_ against TAg is at least an order of magnitude greater than that of the Mcm complexes. In contrast, 314850 preferentially inhibited Mcm2-7 relative to Mcm467 but had little effect on TAg.

### Mechanism of inhibition

**Figure 3:**
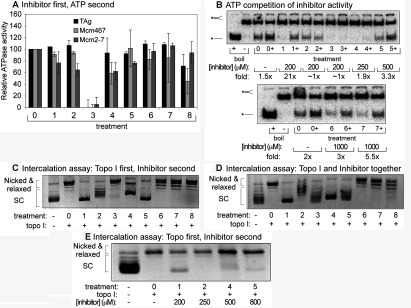
Mode of action of the various small molecule inhibitors Treatment order for each panel: 0, solvent control; 1, compound 125248; 2, 924384; 3, MAL2-11b; 4, 268973; 5, 388612; 6, 314850; 7, 271327; and 8, ciprofloxacin. (**A**) Effects of each inhibitor on steady-state ATP turnover by Mcm2-7, Mcm467 and TAg. This experiment was identical to that shown in Figure 1(C) with the indicated helicase complexes used at 100 nM concentration, but 1 mM of the indicated inhibitor was added prior to ATP addition. Bar graphs show the levels of ATP hydrolysis observed after 30 min of incubation as a % of the ATP hydrolysis observed in the absence of inhibitor (treatment 0). (**B**) Effect of increased ATP concentration with indicated inhibitor on DNA unwinding activity of Mcm2-7. The standard helicase reaction was supplemented with the indicated inhibitor concentration (numbered 1–8 as in A) in the presence (+) or absence of an additional 5 mM ATP. ATP and the indicated inhibitor were added together to Mcm2-7 without preincubation. ‘Fold’ refers to the ratio of DNA unwinding between the reactions containing 10 mM ATP and containing 5 mM ATP. (**C**) Ability of inhibitors to intercalate into DNA. In the intercalation assay (Experimental Procedures), Topo I (4 units) was used to relax 50 ng of monomeric pUC19 (treatment 0; compare supercoiled DNA (left) with relaxed DNA (right)). After 1 h of Topo I treatment, 1mM of the indicated inhibitor was added and samples were incubated for an additional 1 h (D) Topo I activity inhibition assay. This experiment was identical to (**C**), except that Topo I and the indicated inhibitor were added at the same time. Topo I inhibition is indicated if addition of both inhibitor and topoisomerase together generates supercoiled DNA, while experiments shown in (**C**) generate relaxed plasmid. (**E**) An intercalation assay performed with the indicated inhibitors at lower concentrations. These assays were similar to (**C**) (Topo I added first, and the inhibitor added second), except the indicated concentration of inhibitor was used.

As noted above, DNA unwinding is the culmination of a variety of simpler biochemical activities. Thus, the seven representative inhibitors and ciprofloxacin may function by physically interacting with the helicase, the DNA substrate, or the ATP. To understand how all eight inhibitors block helicase activity, their effects on steady-state ATP hydrolysis were measured (Figure 3A). Relative to MAL2-11b, which completely inhibits ATP hydrolysis of Mcm2-7, Mcm467 and TAg [Figure 1C (treatment 7) and 3A (treatment 3)], both the general and Mcm-selective inhibitors demonstrated only a modest inhibition of ATP hydrolysis (e.g., 268973; Figure 3A, treatment 4), while several demonstrated essentially no inhibition of ATP hydrolysis (e.g., 314850 and 271327; Figure 3A, treatments 6 and 7).

These results suggest one of three possible scenarios: First, the inhibitors (with the possible exception of MAL2-11b) might not target the ATPase active sites. Secondly, the inhibitors may deregulate or uncouple the activity of the enzyme rather than block ATP hydrolysis. Thirdly, at least in the case of the Mcm2-7 complex, the inhibitors could preferentially target the ATPase active sites but are selective for the low-turnover regulatory sites. Although the second and third possibilities are difficult to distinguish, the first explanation can be tested. Although we cannot rigorously test for competitive inhibition using our helicase endpoint assay, we can test if increased ATP concentration overcomes the inhibitory effects of these compounds (Figure 3B). Although doubling the ATP concentration in the absence of inhibitor caused a slight increase in helicase activity (1.5- to 2-fold, Figure 3B, treatment 0), in most cases, doubling the ATP concentration in the presence of the inhibitors caused a much larger increase in activity (3- to 20-fold). These results suggest that the inhibitors disrupt ATPase active sites in the Mcm2-7 complex in some manner. In contrast the inhibitory effects of 924384, MAL2-11b, and 268973 could not be rescued by an increase in ATP concentration (Figure 3B, treatments 2-4), suggesting that these inhibitors operate independently of the ATPase active sites.

Because these compounds are also planar double ring molecules, they could conceivably inhibit helicase activity via DNA intercalation. To examine this model, we tested our inhibitors in a standard topoisomerase assay [21]. The rationale of this assay is that intercalating compounds will introduce supercoils into a fully relaxed plasmid. Topo I will remove these introduced supercoils, but after quenching and gel electrophoresis the intercalator will diffuse away and produce a detectable compensatory supercoiling increase.

Following plasmid relaxation, each inhibitor was added to 1 mM final concentration in the topoisomerase assay (Figure 3C, treatments 1–8). The general inhibitors 125248 (treatment 1), 924384 (treatment 2), 268973 (treatment 4) and 388612 (treatment 5) cause extensive DNA intercalation, while in contrast, MAL2-11b (treatment 3) and the more Mcm-selective inhibitors (314850, 271327 and ciprofloxacin, treatments 6-8) demonstrated little or no intercalation (Figure 3C). However, lack of apparent intercalation could also be caused by Topo I inhibition. To test this possibility, the assay was repeated under conditions in which Topo I and each inhibitor were added to the reaction at the same time. Under these conditions, Topo I inhibition will only yield supercoiled plasmids (Figure 3D). Under this criterion and comparing the results to Figure 3C, only MAL2-11b (Figure 3D, treatment 3) is a Topo I inhibitor. Although the general inhibitors can intercalate into dsDNA at 1 mM concentration (Figure 3C), *in vitro* helicase inhibition occurs at much lower inhibitor concentrations. Repeating the intercalation assay at more modest inhibitor concentrations (2- to 3-fold overcalculated IC_50_ for helicase inhibition) only 125248 and 268973 continued to demonstrate significant DNA intercalation (Figure 3E, treatments 1 and 4). Thus, most of the tested inhibitors, including ciprofloxacin, do not appear to function through intercalation, suggesting that they more directly affect the helicase activity.

### Ciprofloxacin preferentially inhibits Mcm2-7 *in vitro* and in yeast and cell culture

An ideal Mcm2-7 inhibitor would specifically target this helicase both biochemically and in living cells. To test this hypothesis, these properties were assayed in the following experiments.

To further define inhibitor selectivity, we examined their *in vitro* effects on representative helicases at 1 mM concentration (Supplementary Figure S3 available at http://www.bioscirep.org/bsr/033/bsr033e072add.htm). Inhibitors 125248, 924384 and 268973 (treatments 1–3) were the least specific, causing nearly complete inhibition of DnaB and T4 gp41. Interestingly, only one additional inhibitor (314850, treatment 6) effectively inhibited the SsoMcm complex. This discrepancy may be due to the high assay temperature (65°C) required to assess SsoMcm helicase activity [25]. Inhibitor 271327 (treatment 7) caused substantially less inhibition among the helicases tested than either 125248 or 924384. In contrast, none of the tested helicases were substantially inhibited by ciprofloxacin (Supplementary Figure 3, treatment 8). Combined with the IC_50_ data summarized in Table 1, Mcm2-7 is the only helicase tested that is preferentially inhibited by ciprofloxacin.

**Figure 4:**
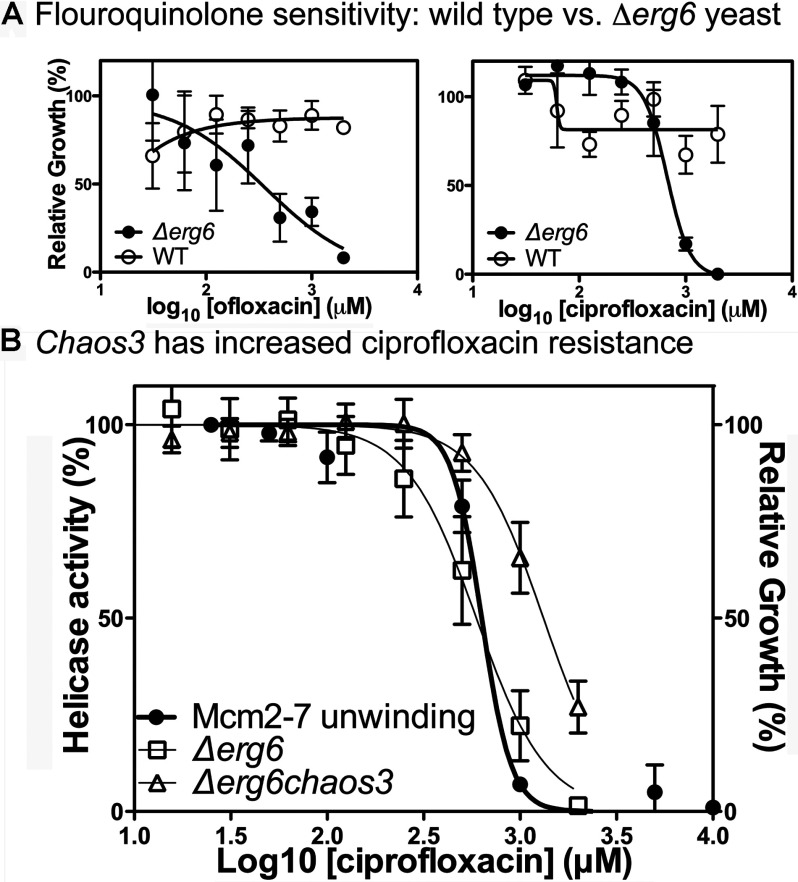
An Mcm mutant demonstrates increased ciprofloxacin resistance (**A**) Effects of the *∆erg6* mutation on growth inhibition of yeast to fluoroquinolones. Cell growth in wild-type and *∆erg6* strains are shown for both ofloxacin (left) and ciprofloxacin (right). (**B**) Inhibition of growth by ciprofloxacin. In yeast, cellular growth and *in vitro* helicase activity is impaired with nearly identical concentration dependence. Mcm4chaos3 mutants demonstrate increased resistance to ciprofloxacin. In all graphs, the data represent the average of ≥3 experiments, and the error bars represent the S.D.

Secondly, to examine the general cellular toxicity of these inhibitors, growth inhibition of micro-cultures by serial dilution of inhibitors was tested in a 96-well format in yeast [22]. Wild-type yeast is resistant to ciprofloxacin (Figure 4A). However, resistance to many compounds in yeast reflects an inability to accumulate sufficient concentrations of such compounds due to the prevalence of multidrug transporters (reviewed in [26]). To circumvent this potential problem, we used a yeast mutant (*∆erg6*) [27] previously shown to non-specifically decrease drug resistance. As anticipated, this strain had demonstrable growth sensitivity to both ofloxacin and ciprofloxacin (Figure 4A).

Using the *∆erg6* strain, the remaining compounds were tested for growth inhibition over a range of concentrations (Supplementary Figure S4 available at http://www.bioscirep.org/bsr/033/bsr033e072add.htm and Table 1). Several compounds inhibited growth at lower concentrations than they inhibited *in vitro* helicase activity (388612, 268973 and 924284), suggesting that proteins other than Mcm2-7 are more sensitive to inhibition. These data are consistent with their poor helicase selectivity as demonstrated above. In contrast, several compounds were less efficient at inhibiting yeast growth than helicase activity (125248 and 314850). However, two inhibitors (ciprofloxacin and to a lesser extent 271327) have IC_50_ curves that closely match the IC50 curves for Mcm2-7 helicase activity (Figure 4B, Table 1), consistent with the possibility that the primary cellular target is Mcm2-7.

Inhibitor cytotoxicity was next examined in a non-tumour human cell line (RPE-TERT, Supplementary Figure S4). In general, these cells were demonstrably more sensitive to the tested inhibitors than yeast. RPE-TERT cells were ~10-fold more sensitive to 125248 and 924384 (IC_50_s of about 10 μM) than 271327 and 314850 (IC_50_s~500–700 μM). The extreme sensitivity of human cells to both 125248 and 924384 suggests that Mcm2-7 is not a major cellular target. In contrast ciprofloxacin kills human cells and inhibits yeast growth at roughly similar concentrations (i.e., human cells are only ~2.5-fold more sensitive than yeast).

### An mcm mutant that confers ciprofloxacin resistance

If Mcm2-7 is an important cellular target of ciprofloxacin, then Mcm mutants should exist that confer ciprofloxacin resistance. We tested a variety of viable Mcm alleles for ciprofloxacin resistance (results not shown) and found one previously studied mutant (*mcm4chaos3* [15]) that was significantly (*P*<0.005) more resistant to ciprofloxacin relative to the *∆erg6* parental strain (Figure 4B) (*∆erg6* IC_50_: 590 μM (95% CI=520–670 μM) against *∆erg6 mcm4chaos3*: 1300 μM (95% CI=1200–1400 μM). Combined with the data described above, we conclude that Mcm2-7 is a ciprofloxacin target.

## DISCUSSION

We provide evidence that ciprofloxacin (and to a lesser extent compound 271327) inhibits the activity of the budding yeast Mcm2-7 helicase both biochemically and in cell culture. Although our experiments largely focus on yeast, we also demonstrated that ciprofloxacin inhibits the viability of human cells at roughly similar concentrations. As fluoroquinolones have been extensively used in human medicine and their pharmacological properties are established [24], the fluoroquinolone scaffold might well serve as a useful platform in the development of Mcm2-7 inhibitors with enhanced therapeutic potential. Although inhibition of Mcm2-7 occurs at ciprofloxacin concentrations higher than its normal therapeutic range (also see below), our results suggest that some of the side effects seen with this and other fluoroquinolones may be because of inhibition of DNA replication.

### Relationship to prior studies

Fluoroquinolones serve as potent antibiotics due to their strong inhibition of the prokaryotic DNA gyrase. Although eukaryotes are relatively resistant to ciprofloxacin at normal therapeutic levels, cytotoxicity is noted at high drug concentrations (reviewed in [24]). The eukaryotic Topo II enzyme is a target for fluoroquinolones such as ciprofloxacin, as the drug inhibits Topo II *in vitro* [28], and mutants in Topo II have been isolated with increased *in vitro* fluoroquinolone resistance [29]. Moreover, cells exposed to cytotoxic levels of fluoroquinolones arrest in G2 and demonstrate chromosomal breaks consistent with the known role of topoisomerase II in mitosis [30]. However, it should be noted that these are also relatively common phenotypes of various known DNA replication mutants (e.g. [31])

Both our *in vitro* and cell-based studies strongly support Mcm2-7 as a new eukaryotic target for fluoroquinolones. Our finding that the Mcm mcm4chaos3 mutant has significantly increased ciprofloxacin resistance provides evidence that at least part of fluoroquinolone cytotoxicity is likely due to defects in DNA replication.

### Inhibitory effects of amino acid modifiers

Although chemically reactive amino acid modifying agents are too unstable, non-specific and irreversible to assist in studies of Mcm2-7 *in vivo*, there is considerable precedence for using modifying reagents *in vitro* to determine a mode of action in complex systems [4]. For example, DNA replication requires a large number of nucleotide hydrolases (e.g., ORC, Cdc6, Mcm2-7, RFC, primase and DNA polymerases [6]), and knowledge of the inhibitory spectrum of modifiers on individual replication factors will aid future studies that examine functional interactions between these proteins. Because preincubation of TAg with ATP relieved much of the inhibitory effects of these modifiers (Figure 1B), they most probably affect ATP binding and oligomerization of TAg, which is ATP-dependent. One interesting difference between inhibition of the Mcms and TAg is with the guanidyl modifier PG, which inhibits both Mcm2-7 and Mcm467 without affecting TAg. This property could make PG an experimentally useful reagent *in vitro* if Mcm2-7 activity needs to be specifically ablated.

### Mode of (fluoro)quinolone inhibition

Our results suggest that most of the studied inhibitors likely interfere with the ATPase active sites of the helicases. Although these molecules only have a modest effect on bulk ATP hydrolysis of Mcm2-7 (Figure 3A), helicase inhibition is largely suppressed by increased ATP concentration (Figure 3B). The relatively high observed IC_50_ concentrations are consistent with this possibility, as the ATP Km_0.5_ for helicase activity by the yeast Mcm2-7 is ~2 mM [19]. However, if (fluoro)quinolones act as inhibitors of ATPase active sites, how can the relatively minor inhibition of ATP hydrolysis be explained?

For Mcm2-7, bulk ATP hydrolysis correlates poorly with DNA unwinding. There are mutations that cause substantial reductions in ATP hydrolysis but have only minor effects on *in vitro* DNA unwinding (e.g., *mcm3KA* [19]), whereas other mutations retain robust steady-state ATP hydrolysis but reduce *in vitro* DNA binding or unwinding (e.g., *mcm6DENQ* [19,32]). Only two of the Mcm2-7 ATPase active sites are responsible for most of the observed steady-state ATP hydrolysis (i.e., the Mcm3/7 and 7/4 active sites [18,33]). The remaining active sites, although clearly essential, hydrolyse ATP poorly. These data suggest that occupancy and turnover at these sites correspond predominately to a regulatory role rather than a direct contribution to helicase function. If the (fluoro)quinolone inhibitors preferentially target the regulatory rather than catalytic sites, only a modest change in ATP hydrolysis might be observed. Alternatively, the inhibitors may function to poison the helicase. By binding to a single active site, the inhibitor might uncouple ATP hydrolysis from DNA unwinding by altering the ability of adjacent active sites to communicate. This model also explains the effect of these inhibitors on TAg, a homohexameric helicase that contains identical ATPase active sites that coordinately unwind DNA during SV40 replication [23]. Finally, the fluoroquinolones could inhibit helicase activity by blocking ssDNA binding; however, this interpretation is difficult to reconcile with our observations that elevated levels of ATP restore Mcm2-7 helicase activity in the presence of most of the examined fluoroquinolones (Figure 3B).

### Prospects for tailoring fluoroquinolones as effective helicase inhibitors for Mcm2-7

Helicases are abundant in eukaryotes. For example, in yeast, ~2% of open reading frames contain known helicase structural motifs [34]. In addition to Mcm2-7, many human helicases (e.g., the RecQ family members such as the Werner, Bloom and RecQ4 helicases, [35]) are also potential therapeutic targets. Given the paucity of available helicase inhibitors and our observations that different fluoroquinolones differentially inhibit a variety of helicases (Supplementary Figure S3), fluoroquinolones may provide a general and malleable molecular scaffold for the development of efficient helicase inhibitors with tailored specificities.

Further development of fluoroquinolones provides a useful route to develop Mcm2-7-specific inhibitors of the therapeutic value, as Mcm overexpression correlates with cancer, and multiple studies indicate that the Mcm2-7 subunits are potential targets [14,36]. Several of the inhibitors that we examined (ciprofloxacin, 271327 and 314850), demonstrate at least partial selectively for Mcm2-7 over a host of other helicases tested and ciprofloxacin appears to target Mcm2-7 in yeast. As ciprofloxacin and related fluoroquinolones are common and approved human antibiotics [37],this molecular scaffold has proven pharmaceutical utility. Although our inhibitors only act at concentrations that exceed typical therapeutic use, this situation has precedence. For example, high doses of sodium phenylbutyrate are used in the treatment of malignant tumours, in which plasma concentrations of the compound are well over 1 mM [38]. Given the degree of selectivity that we observe with an off-the-shelf pharmaceutical designed for an entirely different application, our limited screen of ciprofloxacin-related compounds has identified several chemicals with improved properties, validating the likelihood that additional structural refinement using ciprofloxacin as a starting point will yield molecules with enhanced potency and specificity.

Our discovery of Mcm2-7 inhibitors has utility in other areas. First, they may function as a useful research tool both *in vitro* and *in vivo*. As each of the six Mcm subunits are individually essential, analysis of the role of the replicative helicase has largely focused on model systems such as *S. cerevisiae* that have especially well-developed genetic tools. Such inhibitors also have potential utility for biochemical studies, especially using systems (e.g., *Xenopus* egg extracts [39]) that have highly tractable biochemical advantages but are poorly amenable to genetic manipulation. Secondly, the discovery that fluoroquinolones can inhibit the eukaryotic helicase may explain some of the cytotoxic effects observed with ciprofloxacin and other fluoroquinolones [40]. Our finding that the *mcm4chaos3* allele confers resistance to ciprofloxacin supports our hypothesis that the Mcm2-7 complex is a ciprofloxacin target in cells and suggests that it could also be contributing to the deleterious side effects seen with this class of compounds.

## Online data

Supplementary data
