# Supplementary material for: Ciprofloxacin is an inhibitor of the Mcm2-7 replicative helicase
Source: Biosci Rep. 2013 Oct 7;33(5):e00072. doi: 10.1042/BSR20130083 (PMC3791872; doi:10.1042/BSR20130083)
Supplement: Supplementary data [file bsr033e072add.pdf]

Biosci. Rep. (2013) / **33** / art:e00072 / doi 10.1042/BSR20130083

OPEN ACCESS

Original Paper

SUPPLEMENTARY DATA

Ciprofloxacin is an inhibitor of the Mcm2-7 replicative helicase

Nicholas SIMON*[1], Matthew L. BOCHMAN*‡[1], Sandlin SEGUIN*, Jeffrey L. BRODSKY*, William L. SEIBEL† and Anthony SCHWACHA*[2]

*Department of Biological Sciences, University of Pittsburgh, Pittsburgh, PA 15260, U.S.A., †Drug Discovery Center, University of Cincinnati, Cincinnati, OH 45237, U.S.A., and ‡Molecular and Cellular Biochemistry Department, Indiana University, Bloomington, IN 47405, U.S.A.

MATERIALS AND METHODS

The viability of human cells was assayed using the MTS method [1]. Briefly, 1×10^5 cells of the human non-tumour cell line RPE-hTERT were plated into each well of a 96-well plate and grown in DMEM: F12 containing 10 % (v/v) FBS in 5 % (v/v) CO_2 at 37 °C. The next day, the indicated compounds were titrated into media such that the final concentration contained 1 % (v/v) DMSO. As a negative control, media were also prepared that contained 1 % DMSO but lacked compound. After 48 h, the media was removed and replaced with DMEM lacking phenol red but containing Cell Titer 96 Aqueous One Solution Cell Proliferation Assay (Promega). After 1 h, the $A_{490\ nm}$ was measured using a BioRad iMark Microplate Reader (Hercules). Final data reflect the average and standard deviation (S.D.) of three replicates at each compound concentration.

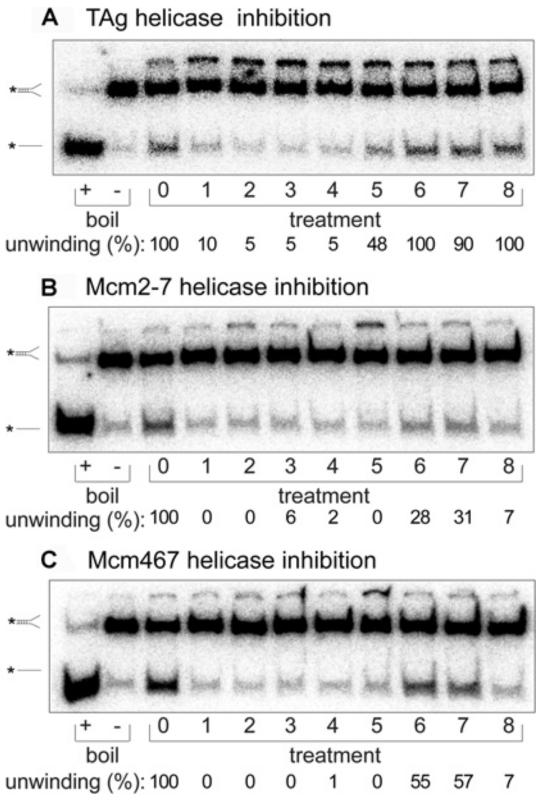

Figure S1 Effects of select (fluoro)quinolone inhibitors on (A) TAg helicase, (B) Mcm2-7 and (C) Mcm467activity

For each panel: +, boiled DNA fork; -, intact fork; 0, solvent control; 1, compound 125248; 2, 924384; 3, MAL2–11b; 4, 268973; 5, 388612; 6, 314850; 7, 271327; and 8, ciprofloxacin. Inhibitors were pre-incubated with the indicated helicase at a final concentration of 1 mM, as in Figure 1. The inhibitors were preincubated with helicase before ATP addition, and the final helicase concentration in all experiments was 100 nM (hexamer). The values below the gels indicate the percent of DNA unwinding by the indicated helicase normalized to the solvent control (treatment 0).

[1] These authors contributed equally to this work.

[2] To whom correspondence should be addressed (email schwacha@pitt.edu).

© 2013 The Author(s) This is an Open Access article distributed under the terms of the Creative Commons Attribution Licence (CC-BY) (http://creativecommons.org/licenses/by/3.0/) which permits unrestricted use, distribution and reproduction in any medium, provided the original work is properly cited.

Bioscience Reports www.bioscirep.org

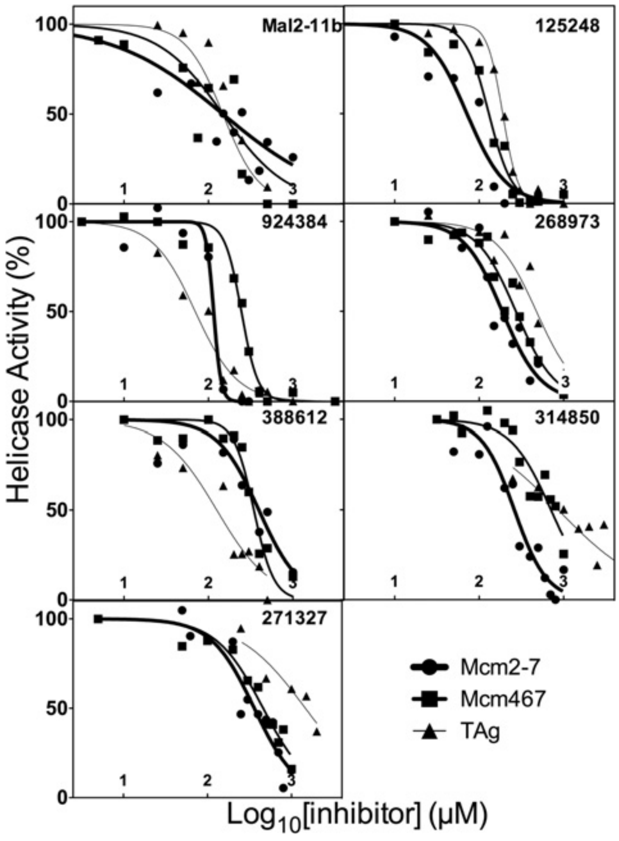

Figure S2 The identified inhibitors exhibit diverse specificities against different helicases

Representative helicase activity assays in the presence of the indicated inhibitor were tested, quantified and standardized as described in the legend to Figures 2 (A) and (B). All helicases were assayed at 100 nM final concentration (hexamer) with inhibitor preincubation prior to ATP addition.

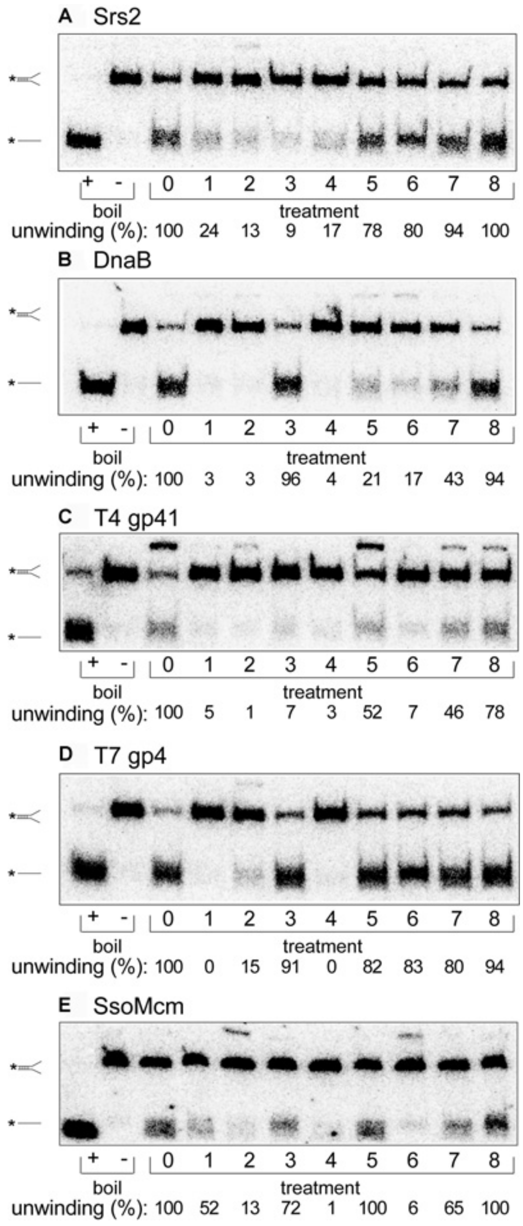

Figure S3 Ciprofloxacin poorly inhibits hexameric helicases unrelated to the Mcms

All inhibitors were used at 1 mM final concentration. Lane order for each panel: +, boiled DNA fork; -, intact fork; 0, solvent control; 1, compound 125248; 2, 924384; 3, MAL2–11b; 4, 268973; 5, 388612; 6, 314850; 7, 271327; and 8, ciprofloxacin. The helicase tested is listed at the top of each gel, and the percent of helicase activity remaining in the presence of inhibitor is listed below each gel. The reaction conditions used for each helicase are similar to that used for Mcm2-7 and described in the Experimental Procedures. The values below the gels indicate the per cent of DNA unwound by the indicated helicase normalized to the solvent control (treatment 0). All helicases were used at 100 nM concentration (hexamer) with inhibitor pre-incubation as described in the Materials and Methods section.

© 2013 The Author(s) This is an Open Access article distributed under the terms of the Creative Commons Attribution Licence (CC-BY) (http://creativecommons.org/licenses/by/3.0/) which permits unrestricted use, distribution and reproduction in any medium, provided the original work is properly cited.

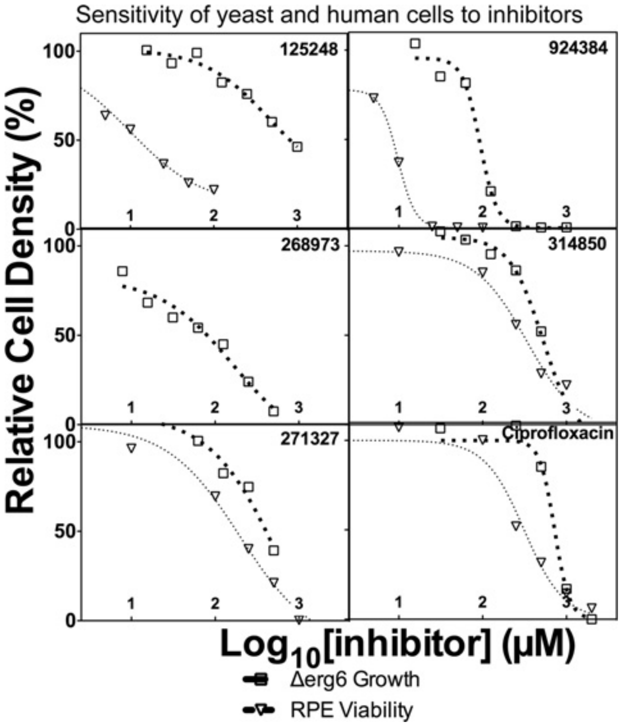

Figure S4 Effects of Mcm inhibitors on yeast growth and human cell viability
Representative assays are shown as described in the Experimental Procedures and Supplementary Methods, and the results are normalized to growth in the presence of 1 % (v/v) DMSO.

© 2013 The Author(s) This is an Open Access article distributed under the terms of the Creative Commons Attribution Licence (CC-BY) (http://creativecommons.org/licenses/by/3.0/) which permits unrestricted use, distribution and reproduction in any medium, provided the original work is properly cited.

TABLE S1 The following chemical compounds were added to helicase assay of Mcm2-7, Mcm467 and SV-40 T antigen at 1 mM final concentration

The values listed are per cent inhibition relative to the activity of each enzyme in the absence of inhibitor. Names in bold denote compounds of interest that inhibited activity by >90 %. Values listed are the average of at least three experimental repeats with the corresponding S.D.

Structures	Name	Mcm2-7	Mcm467	TAg
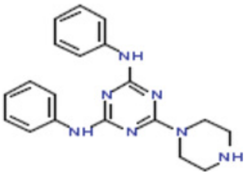	**924384**	0 ± 0	1.3 ± 1.3	10.6 ± 5
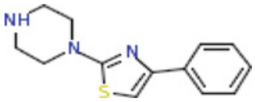	981780	54 ± 25	106 ± 6	104 ± 18
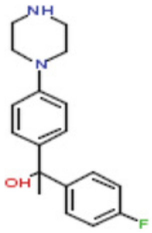	155971	54 ± 6	32 ± 6	90 ± 16
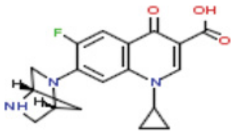	102362	40 ± 10	40 ± 2	92 ± 5
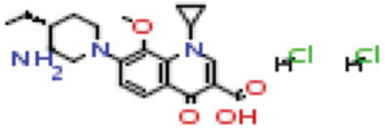	454789	71 ± 5	41 ± 16	101 ± 16
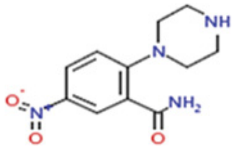	939001	53 ± 2	49 ± 20	81 ± 2
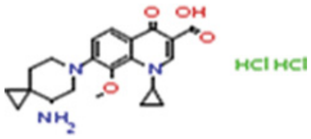	441520	47 ± 14	36 ± 9	78 ± 0.3
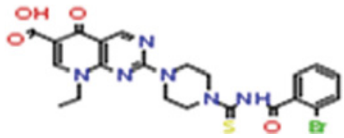	780771	79 ± 14	55 ± 2	89 ± 8
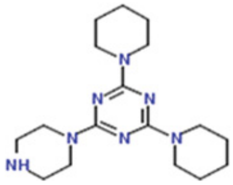	921213	47 ± 13	79 ± 17	100 ± 13

© 2013 The Author(s) This is an Open Access article distributed under the terms of the Creative Commons Attribution Licence (CC-BY) (http://creativecommons.org/licenses/by/3.0/) which permits unrestricted use, distribution and reproduction in any medium, provided the original work is properly cited.

TABLE S1 Continued

Structures	Name	Mcm2-7	Mcm467	TAg
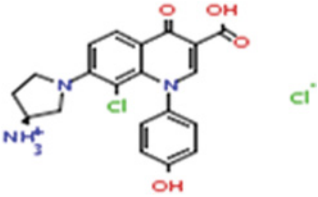	**268973**	0 ± 0	11 ± 5	26 ± 5
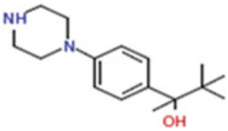	**155968**	13 ± 8	8 ± 8	71 ± 4
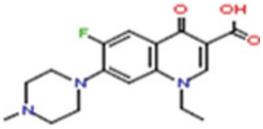	99564	22 ± 4	34 ± 1	74 ± 15
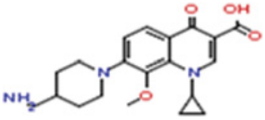	469514	40 ± 17	24 ± 3	91 ± 16
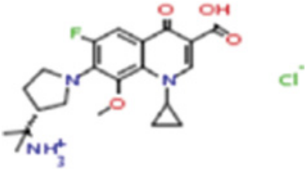	358088	50 ± 0.5	14 ± 4	79 ± 6
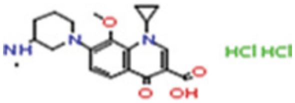	311135	31 ± 9	3 ± 3	29 ± 2
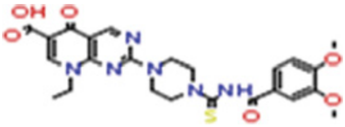	780938	40 ± 18	29 ± 16	97 ± 13
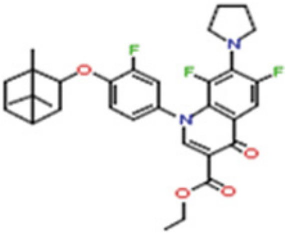	414145	37 ± 11	46 ± 6	106 ± 8
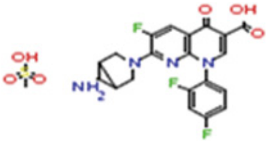	177528	33 ± 9	7 ± 1	86 ± 3
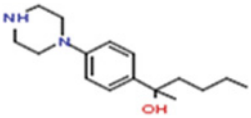	155975	44 ± 5	42 ± 19	95 ± 6

© 2013 The Author(s) This is an Open Access article distributed under the terms of the Creative Commons Attribution Licence (CC-BY) (http://creativecommons.org/licenses/by/3.0/) which permits unrestricted use, distribution and reproduction in any medium, provided the original work is properly cited.

TABLE S1 Continued

Structures	Name	Mcm2-7	Mcm467	TAg
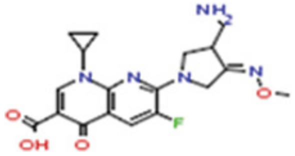	787796	36 ± 8	29 ± 29	117 ± 14
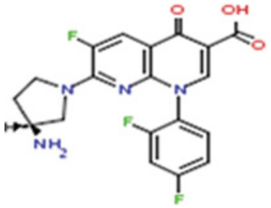	102328	28 ± 17	32 ± 17	111 ± 7
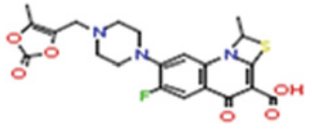	694829	40 ± 9	20 ± 1	100 ± 6
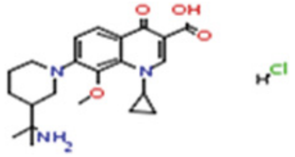	**354880**	0 ± 0	4 ± 4	28 ± 9
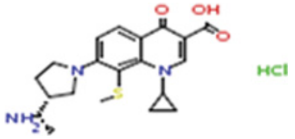	**314850**	0 ± 0	18 ± 18	84 ± 13
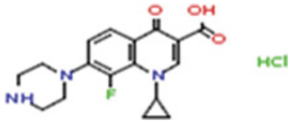	117756	52 ± 19	53 ± 30	94 ± 1
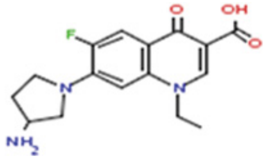	**101683**	22 ± 8	58 ± 1	75 ± 7
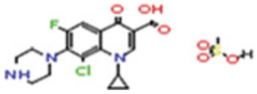	252474	43 ± 14	57 ± 29	96 ± 1
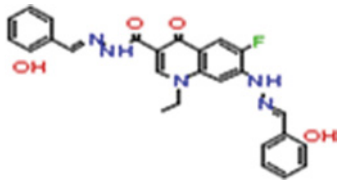	**407174**	0 ± 0	0 ± 0	57 ± 12
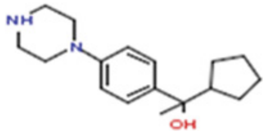	155969	40 ± 14	48 ± 18	78 ± 18

© 2013 The Author(s) This is an Open Access article distributed under the terms of the Creative Commons Attribution Licence (CC-BY) (http://creativecommons.org/licenses/by/3.0/) which permits unrestricted use, distribution and reproduction in any medium, provided the original work is properly cited.

TABLE S1 Continued

Structures	Name	Mcm2-7	Mcm467	TAg
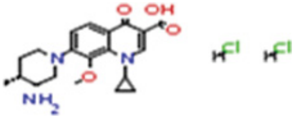	**437813**	71 ± 6	65 ± 26	97 ± 6
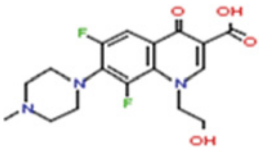	102619	42 ± 7	64 ± 1	99 ± 1
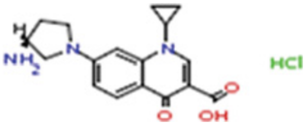	191465	33 ± 16	39 ± 7	56 ± 2
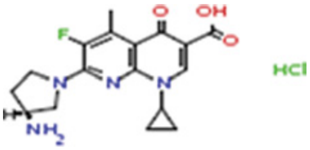	**125248**	0*	5 ± 5	13 ± 4
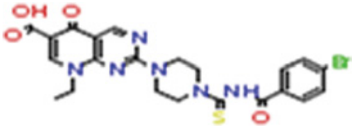	776386	68 ± 19	79 ± 1	71 ± 24
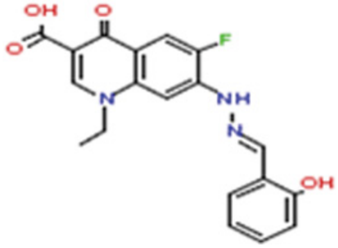	407895	88 ± 4	51 ± 16	86 ± 18
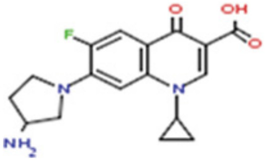	101684	34 ± 5	63 ± 22	84 ± 19
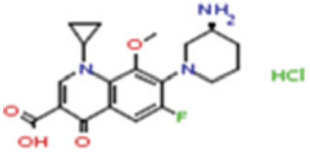	441478	27 ± 12	49 ± 19	87 ± 6
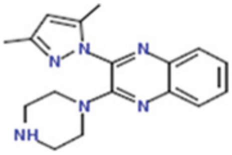	952880	21 ± 3	52 ± 8	79 ± 15
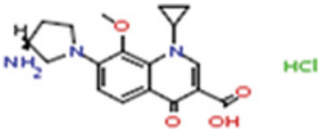	169049	10 ± 6	29 ± 7	66 ± 4

© 2013 The Author(s) This is an Open Access article distributed under the terms of the Creative Commons Attribution Licence (CC-BY) (http://creativecommons.org/licenses/by/3.0/) which permits unrestricted use, distribution and reproduction in any medium, provided the original work is properly cited.

TABLE S1 Continued

Structures	Name	Mcm2-7	Mcm467	TAg
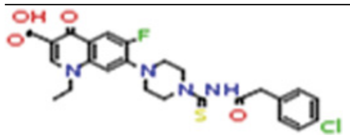	776390	54±31	71±16	94±11
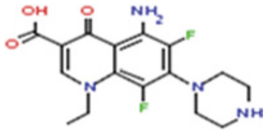	271327	7±3	75±13	89±8
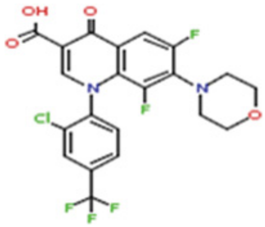	412617	46±6	72±23	83±18
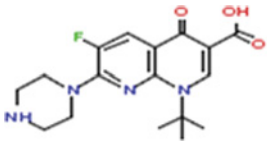	102530	30±3	52±8	83±27
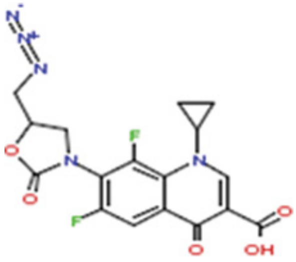	102494	42±10	28±7	83±8
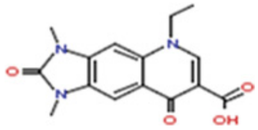	99547	38±25	31±2	99±1
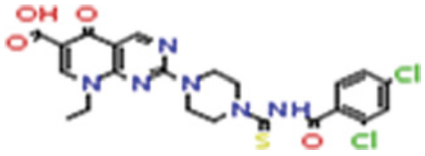	776387	36±10	78±1	96±7
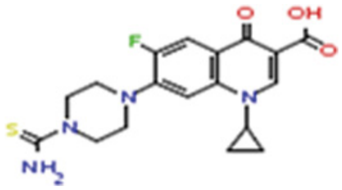	102288	8±8	48±5	93±5
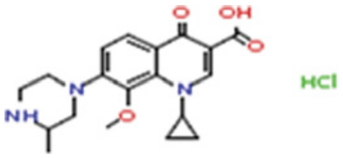	299587	72±48	56±24	87±10

© 2013 The Author(s) This is an Open Access article distributed under the terms of the Creative Commons Attribution Licence (CC-BY) (http://creativecommons.org/licenses/by/3.0/) which permits unrestricted use, distribution and reproduction in any medium, provided the original work is properly cited.

TABLE S1 Continued

Structures	Name	Mcm2-7	Mcm467	TAg
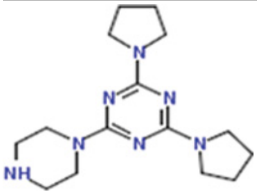	1002635	41±27	75±3	94±2
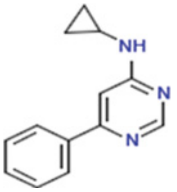	749948	51±35	38±12	83±10
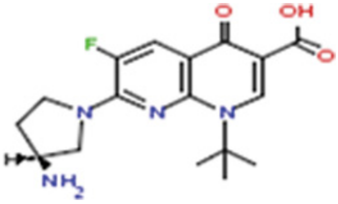	102519	25±4	25±10	95±4
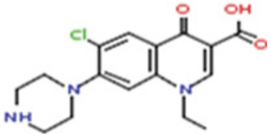	100236	27±7	37±3	95±5
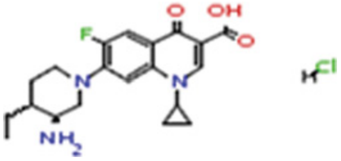	481427	14±7	31±13	102±13
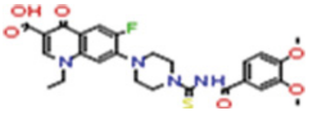	776388	23±11	42±3	101±4
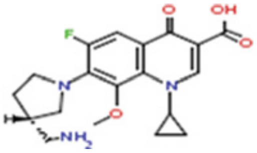	117757	23±1	53±25	95±7
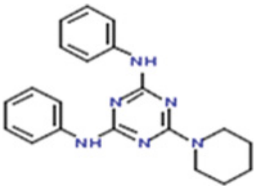	390077	9±1	29±9	40±28
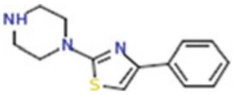	981780	23±6	63±37	101±10
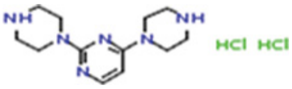	**130040**	0±0	13±13	92±5

© 2013 The Author(s) This is an Open Access article distributed under the terms of the Creative Commons Attribution Licence (CC-BY) (http://creativecommons.org/licenses/by/3.0/) which permits unrestricted use, distribution and reproduction in any medium, provided the original work is properly cited.

TABLE S1 Continued

Structures	Name	Mcm2-7	Mcm467	TAg
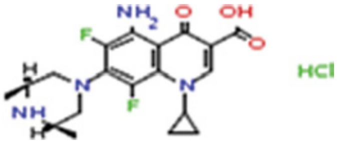	102522	40 ± 12	37 ± 1	82 ± 15
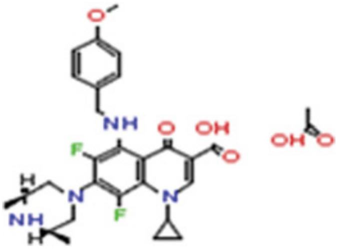	102467	67 ± 38	65 ± 26	99 ± 8
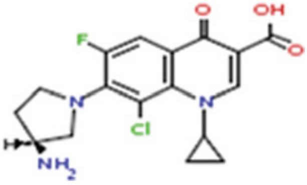	114703	60 ± 40	49 ± 7	108 ± 13
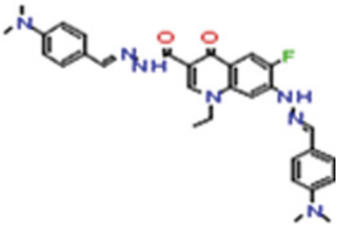	401677	49 ± 23	56 ± 11	104 ± 8
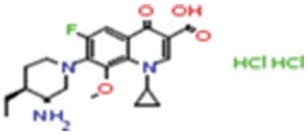	486364	75 ± 41	28 ± 13	54 ± 23
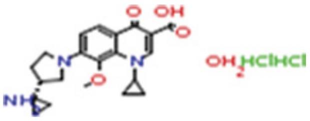	787793	60 ± 11	80 ± 20	102 ± 8
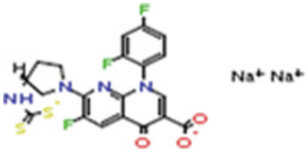	102451	50 ± 18	22 ± 18	85 ± 2
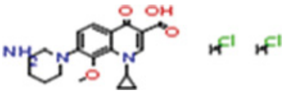	301696	40 ± 25	39 ± 7	110 ± 13
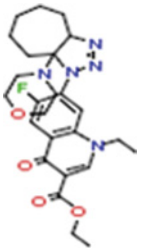	502404	35 ± 9	32 ± 3	94 ± 6

© 2013 The Author(s) This is an Open Access article distributed under the terms of the Creative Commons Attribution Licence (CC-BY) (http://creativecommons.org/licenses/by/3.0/) which permits unrestricted use, distribution and reproduction in any medium, provided the original work is properly cited.

TABLE S1 Continued

Structures	Name	Mcm2-7	Mcm467	TAg
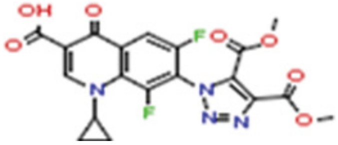	502395	46±22	27±15	88±12
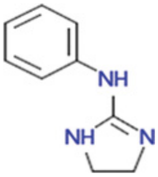	180340	35±7	34±17	100±1
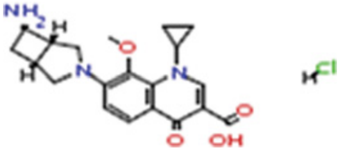	422271	42±26	29±18	83±5
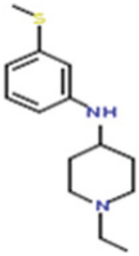	383399	62±14	31±19	97±6
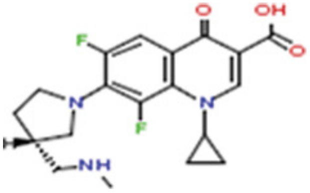	116029	30±2	28±9	119±9
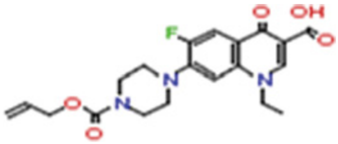	101551	30±1	22±0.5	109±10
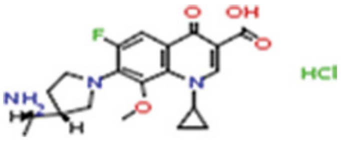	118606	49±22	54±20	100±4
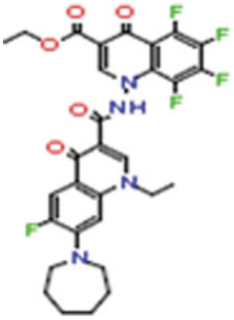	502394	48±32	22±5	85±9

© 2013 The Author(s) This is an Open Access article distributed under the terms of the Creative Commons Attribution Licence (CC-BY) (http://creativecommons.org/licenses/by/3.0/) which permits unrestricted use, distribution and reproduction in any medium, provided the original work is properly cited.

TABLE S1 Continued

Structures	Name	Mcm2-7	Mcm467	TAg
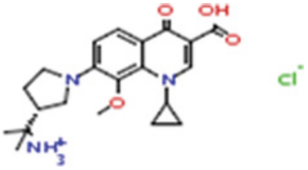	269710	22 ± 8	26 ± 9	108 ± 21
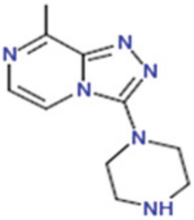	612335	33 ± 19	32 ± 14	108 ± 4
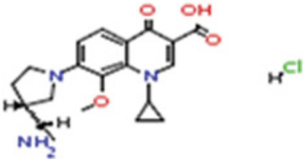	142744	48 ± 23	24 ± 1	108 ± 20
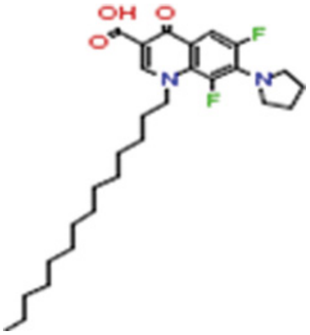	413586	34 ± 10	26 ± 8	97 ± 2
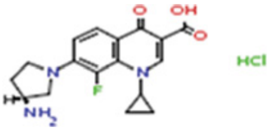	125251	38 ± 15	35 ± 11	101 ± 13
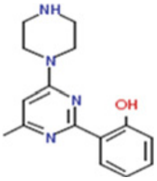	388520	35 ± 6	24 ± 7	97 ± 4
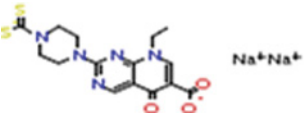	101609	46 ± 11	16 ± 4	100 ± 10
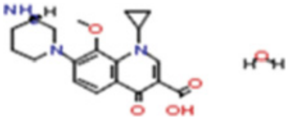	532969	83 ± 45	18 ± 3	82 ± 19
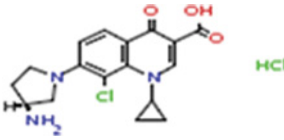	116997	36 ± 9	26 ± 15	78 ± 16

© 2013 The Author(s) This is an Open Access article distributed under the terms of the Creative Commons Attribution Licence (CC-BY) (http://creativecommons.org/licenses/by/3.0/) which permits unrestricted use, distribution and reproduction in any medium, provided the original work is properly cited.

TABLE S1 Continued

Structures	Name	Mcm2-7	Mcm467	TAg
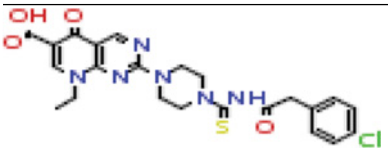	773537	28±17	36±6	72±4
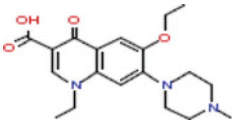	502424	78±52	28±7	54±15
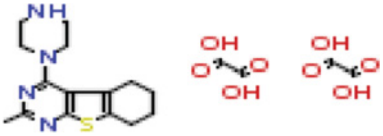	935699	43±33	58±11	56±15
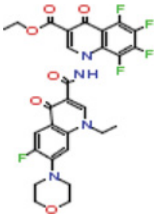	537947	72±4	20±19	79±1
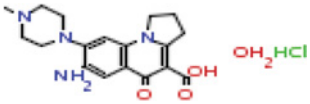	**394299**	9±9	3±3	93±1
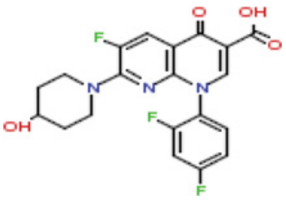	102554	60±9	60±31	95±8
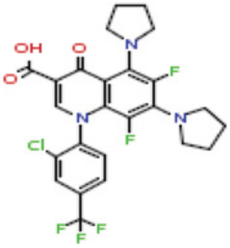	412160	0±0	38±5	75±16
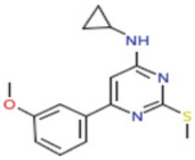	804270	25±2	19±8	82±20

© 2013 The Author(s) This is an Open Access article distributed under the terms of the Creative Commons Attribution Licence (CC-BY) (http://creativecommons.org/licenses/by/3.0/) which permits unrestricted use, distribution and reproduction in any medium, provided the original work is properly cited.

TABLE S1 Continued

Structures	Name	Mcm2-7	Mcm467	TAg
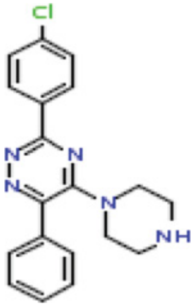	587706	10 ± 7	54 ± 6	64 ± 22
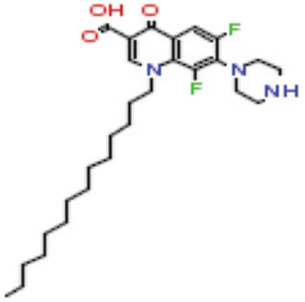	414908	25 ± 3	39 ± 9	62 ± 26
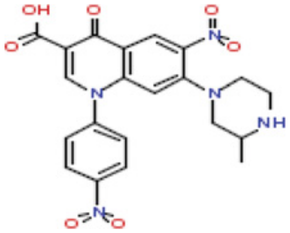	408743	66 ± 1	13 ± 5	78 ± 22
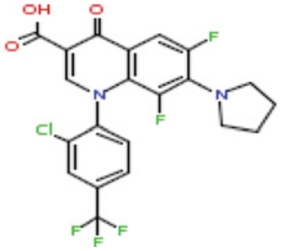	**412152**	10 ± 10	24 ± 5	54 ± 1
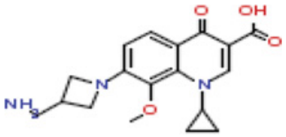	299588	25 ± 5	67 ± 29	82 ± 10
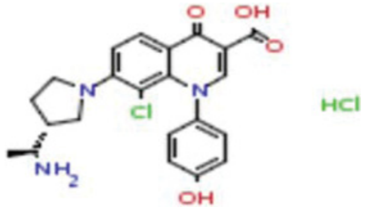	**486369**	0 ± 0	0 ± 0	4 ± 4

© 2013 The Author(s) This is an Open Access article distributed under the terms of the Creative Commons Attribution Licence (CC-BY) (http://creativecommons.org/licenses/by/3.0/) which permits unrestricted use, distribution and reproduction in any medium, provided the original work is properly cited.

TABLE S1 Continued

Structures	Name	Mcm2-7	Mcm467	TAg
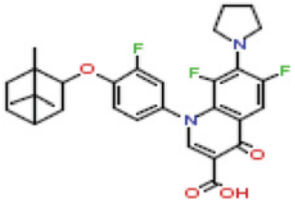	502423	41 ± 25	6 ± 6	93 ± 8
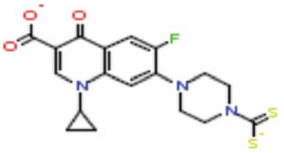	779984	53 ± 36	29 ± 2	89 ± 7
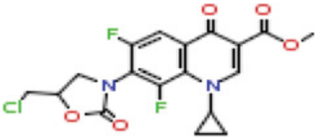	102443	28 ± 15	25 ± 4	72 ± 27
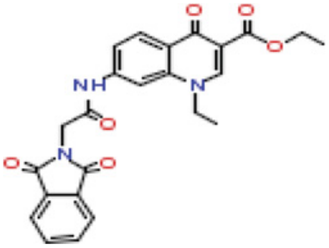	**99540**	2 ± 0.5	18 ± 1	87 ± 1
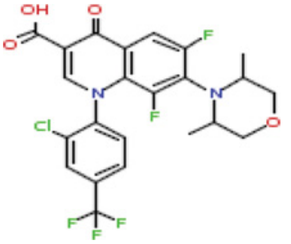	412153	65 ± 34	21 ± 8	88 ± 8
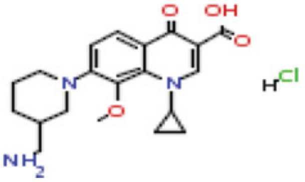	323801	16 ± 1	36 ± 3	87 ± 5
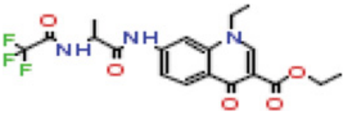	99539	19 ± 18	27 ± 7	74 ± 5
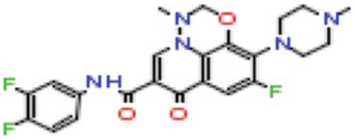	**502433**	12 ± 17	21 ± 7	88 ± 6

© 2013 The Author(s) This is an Open Access article distributed under the terms of the Creative Commons Attribution Licence (CC-BY) (http://creativecommons.org/licenses/by/3.0/) which permits unrestricted use, distribution and reproduction in any medium, provided the original work is properly cited.

TABLE S1 Continued

Structures	Name	Mcm2-7	Mcm467	TAg
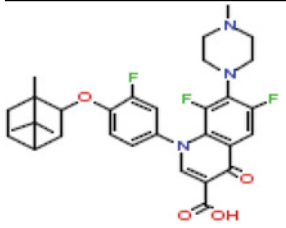	502430	71 ± 42	35 ± 4	85 ± 11
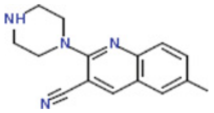	406185	101 ± 40	31 ± 17	95 ± 5
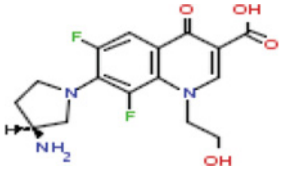	102592	67 ± 31	19 ± 12	81 ± 6
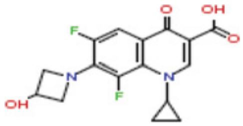	102582	40 ± 40	58 ± 35	94 ± 3
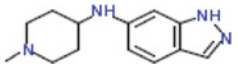	**384191**	0 ± 0	22 ± 8	48 ± 14
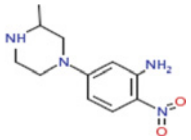	388867	48 ± 8	59 ± 17	94 ± 9
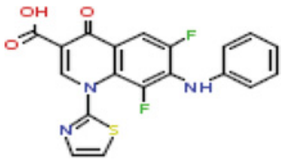	502400	50 ± 20	28 ± 1	99 ± 3
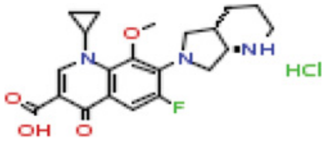	700762	33 ± 13	63 ± 1	92 ± 10
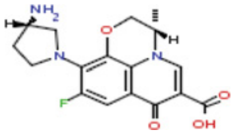	**102650**	3 ± 3	33 ± 1	74 ± 2
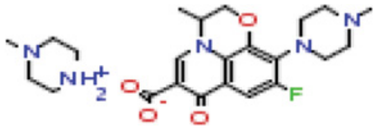	125070	35 ± 16	51 ± 29	84 ± 18
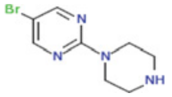	943410	41 ± 15	71 ± 8	95 ± 8

© 2013 The Author(s) This is an Open Access article distributed under the terms of the Creative Commons Attribution Licence (CC-BY) (http://creativecommons.org/licenses/by/3.0/) which permits unrestricted use, distribution and reproduction in any medium, provided the original work is properly cited.

TABLE S1 Continued

Structures	Name	Mcm2-7	Mcm467	TAg
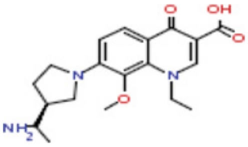	363957	46 ± 27	50 ± 12	104 ± 5
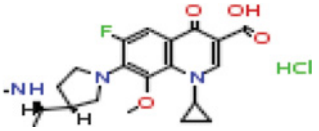	125261	124 ± 47	66 ± 18	100 ± 6
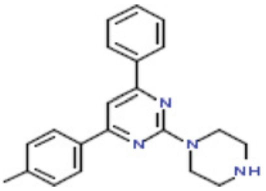	**388612**	0*	25 ± 18	39 ± 11
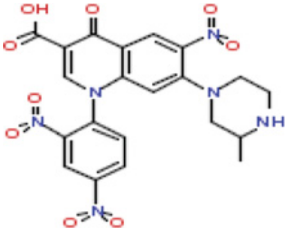	**415275**	17 ± 22	11 ± 9	45 ± 12
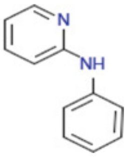	114186	88 ± 28	65 ± 11	92 ± 8

REFERENCE

1 Wright, C. M., Chovatiya, R. J., Jameson, N. E., Turner, D. M., Zhu, G., Werner, S., Huryn, D. M., Pipas, J. M., Day, B. W., Wipf, P. et al. (2008) Pyrimidinone–peptoid hybrid molecules with distinct effects on molecular chaperone function and cell proliferation. Bioorg. Med. Chem. **16**, 3291–3301

Received 24 July 2013/20 August 2013; accepted 3 September 2013

Published as Immediate Publication 3 September 2013, doi 10.1042/BSR20130083

© 2013 The Author(s) This is an Open Access article distributed under the terms of the Creative Commons Attribution Licence (CC-BY) (http://creativecommons.org/licenses/by/3.0/) which permits unrestricted use, distribution and reproduction in any medium, provided the original work is properly cited.
